# Diagnosis of Bleeding Meckel's Diverticulum in Adults

**DOI:** 10.1371/journal.pone.0162615

**Published:** 2016-09-14

**Authors:** Sung Noh Hong, Hyun Joo Jang, Byong Duk Ye, Seong Ran Jeon, Jong Pil Im, Jae Myung Cha, Seong-Eun Kim, Soo Jung Park, Eun Ran Kim, Dong Kyung Chang

**Affiliations:** 1 Department of Internal Medicine, Samsung Medical Center, Sungkyunkwan University School of Medicine, Seoul, Republic of Korea; 2 Dongtan Sacred Heart Hospital, Hallym University College of Medicine, Hwaseong, Republic of Korea; 3 Asan Medical Center, University of Ulsan College of Medicine, Seoul, Republic of Korea; 4 Institute for Digestive Research, Soonchunhyang University College of Medicine, Seoul, Republic of Korea; 5 Department of Internal Medicine and Liver Research Institute, Seoul National University College of Medicine, Seoul, Republic of Korea; 6 Gang Dong Kyung Hee University Hospital, Kyung Hee University College of Medicine, Seoul, Republic of Korea; 7 Ewha Womans University College of Medicine, Seoul, Republic of Korea; 8 Institute of Gastroenterology, Yonsei University College of Medicine, Seoul, Republic of Korea; University Hospital Llandough, UNITED KINGDOM

## Abstract

**Background and Aims:**

Various modalities have been used to diagnose Meckel's diverticulum (MD) in practice, but with their diagnostic accuracy deemed to be unsatisfactory for clinical practice. Moreover, the usefulness of these modalities has not been evaluated for the diagnosis of bleeding MD in adults, due to the relative rarity of this condition. Therefore, the aim of our multicenter study was to determine the most accurate modality for the preoperative diagnosis of bleeding MD in adults.

**Methods:**

We conducted a retrospective analysis of the diagnostic accuracy for small bowel bleeding associated with MD of different modalities in patients ≥18 years old who underwent assessment for MD, with confirmation at the time of explorative surgery. Diagnostic accuracy of the different modalities was evaluated against the diagnosis obtained using technetium-99m pertechnetate scintigraphy (also known as Meckel's scan), considered to be the gold standard for the diagnosis of bleeding MD in pediatrics.

**Results:**

Thirty-five adults were identified with bleeding in MD over the study period, between 2005 and 2012. Among these patients, only 24 (68.6%) were diagnosed with MD preoperatively. The mean (95% confidence interval) diagnostic accuracy of selected modalities was as follows: Meckel’s scan, 21.4% (5.7%-51.2%); capsule endoscopy, 35.7% (14.0%-64.4%); balloon-assisted enteroscopy (BAE), 85.0% (61.1%-96.0%); angiography, 0.0% (0.0%-80.2%); computed tomography, 31.8% (14.7%-54.9%); and small-bowel follow-through, 62.5% (25.9%-90.0%). The diagnostic accuracy was significantly higher for BAE than for Meckel’s scan (*P* = 0.001).

**Conclusions:**

Among available diagnostic modalities, BAE provides the highest accuracy for the diagnosis of bleeding MD in adults and, therefore, should be considered as the preferred modality for preoperative diagnosis.

## Introduction

Meckel’s diverticulum (MD) forms part of the differential diagnosis of small bowel bleeding. However, the diagnosis of MD can be difficult, resulting in a delayed or even missed diagnosis [1, 2]. Technetium-99m pertechnetate scintigraphy, commonly known as Meckel’s scan, is considered as the modality of choice to evaluate patients with suspected MD, based on its diagnostic accuracy of approximately 90% in pediatric patients [3]. However, a diagnostic accuracy of <50% has been reported when Meckel’s scan is used in adults [1, 4]. Therefore, various modalities have been adopted for the diagnosis of MD in adults, with the diagnostic accuracy often considered to be unsatisfactory for clinical practice. Moreover, the diagnostic yield and accuracy of these modalities for the diagnosis of bleeding MD, a relatively rare condition, have not been comprehensively evaluated.

Recently, balloon-assisted enteroscopy (BAE) has increasingly been used, providing endoscopic visualization of small bowel pathologies. Given that most MDs are located within 100 cm of the ileocecal valve (ICV) [5], a retrograde BAE approach could feasibly reach MD lesions and, therefore, might provide an effective diagnostic tool for MD [6]. Therefore, the aim of our multicenter study was to evaluate the diagnostic yield and accuracy of different modalities, including BAE, for the diagnosis of bleeding MD in adults, and to use the outcome of our evaluation to determine which diagnostic modality would be most useful for the preoperative diagnosis of bleeding MD in adults.

## Patients and Methods

We undertook a multi-center retrospective analysis of the accuracy of six different modalities for the diagnosis of small bowel bleeding in adult patients. For all patients included in the analysis, MD was confirmed during exploratory surgery. The study period extended from 2005, when BAE was first available in Korea, to 2012. Eligible patients were assessed at the following eight tertiary centers affiliated to our Small Intestine Research Group of the Korean Association for the Study of Intestinal Diseases: Samsung Medical Center, Asan Medical Center, Hangang Sacred Heart Hospital, Soonchunhyang University Seoul Hospital, Kyung Hee University Hospital at Gangdong, Seoul National University Hospital, Severance Hospital, and Konkuk University Medical Center.

Included in our study were consecutive patients, ≥18 years old, with small bowel bleeding in whom the locus of the bleeding could not be identified by conventional esophagogastroduodenoscopy and colonoscopy. Therefore, exploratory laparotomy or laparoscopy was performed to confirm the diagnosis of MD and to locate the locus of bleeding. Patients in whom MD was suspected on imaging but not confirmed by surgery or in whom MD was incidentally identified during abdominal surgery were excluded. This study was approved by the institutional review board of Samsung Medical Center. The records and information were anonymized and de-identified prior to analysis.

### Diagnostic modalities used to detect MD in adult patients with small bowel bleeding

Explorative surgery is the *gold standard* for diagnosis of MD in adult patients, with any symptomatic MD surgically resected. All patients included in our study had undergone at least one or more of the following diagnostic tests to evaluate the cause of small bowel bleeding: Meckel’s scans, capsule endoscopy (CE), BAE, mesenteric angiography, computed tomography (CT) of the abdomen and pelvis, small bowel follow-through (SBFT), and Technetium-99m red blood cell scintigraphy (RBC scan). For each modality, the diagnosis of MD was made according to the following specific criteria: focal bleeding activity in the mid or lower abdomen on Meckel’s scanning [7]; double-lumen sign on CE, defined by a partial disappearance of the normal or ectopic gastric mucosa in the small intestine, or capsule retention in an abnormal blind-ending section of the small bowel [8]; double-lumen sign and/or concurrent ulcerative lesions in the lumen on BAE [1]; extravasation of the contrast material in patients with an actively bleeding rate >0.5 mL/min on mesenteric angiography [9]; blind-ending fluid- or gas-filled structure that is continuous with the small bowel on CT of the abdomen and pelvis [9]; a blind-ending pouch on the antimesenteric side of the distal ileum on barium studies, including SBFT [9]; and abnormal flow of tracer in the right lower abdomen or pelvis and delayed static images showing tracer moving distally in the intestine on RBC scan [10]. During open or laparoscopic surgery, MD was confirmed by the presence of diverticulum in the small bowel, arising from the anti-mesenteric border, a part of the intestine that lies opposite to the mesenteric attachment. In 90% of cases, MD lesions are identified within 90 cm of the ICV, although reports of MDs located up to 180 cm from the ICV have occurred [11]. In most cases, the blood supply to the MD is provided via a terminal branch of the superior mesenteric artery that crosses the ileum to the diverticulum [11, 12].

### Diagnostic yield and accuracy

The diagnosis of MD was classified as follows: *certain MD*, defined as a confirmatory diagnosis of MD based on the findings of a particular diagnostic modality; *presumptive MD*, defined as a diagnosis of MD based on reasonable conclusions from the findings of a particular diagnostic modality; and *other significant findings*, referring to findings detected in the small bowel by a diagnostic modality that were not compatible with the definitions of certain or presumptive MD, but were of sufficient importance to warrant further investigation.

The diagnostic yield for each evaluation modality was defined as the ratio of the number of patients with *certain* or *presumptive* MD, as well as those with *other significant findings*. Generally, accuracy refers to the ability of a modality to actually measure what it claims to measure. Therefore, the diagnostic accuracy of each evaluation modality was defined as the proportion of patients with *certain* or *presumptive* MD only. Both the diagnostic yields and accuracy of the diagnostic modalities were reported by their 95% confidence intervals (95% CI).

### Statistical analysis

A two-sample test was used to evaluate the diagnostic yield and accuracy of an evaluation modality relative to values obtained using Meckel’s scan. A two-tailed *p*-value<0.05 was considered statistically significant. All analyzes were performed with MedCalc software, Version 11.6 (MedCalc, Mariakerke, Belgium).

## Results

A total of 35 adults with bleeding MD were identified between 2005 and 2012. The mean age of these patients was 37.2±15.7 years, with 25 patients (71.4%) being male (Table 1). Among these 35 patients, 24 (68.6%) were diagnosed preoperatively with *certain* or *presumptive* MD. Meckel’s scan was performed in 14 patients, with MD diagnosed in 3 of these patients. The histamine type-2 (H2) receptor antagonist prior to Meckel’s scan was not administrated. CE was also performed in 14 patients, with MD diagnosed in 5 patients and *significant other findings* identified in another 6 patients. BAE was performed in 20 patients, with MD diagnosed in 17 patients and *significant other findings* identified in another 2 patients. Mesenteric angiography was performed in 2 patients, with no diagnosis of MD but with *other significant findings* identified in 1 patient. Abdominal and pelvic CT imaging was performed in 22 patients, with 7 patients diagnosed with MD and *other significant findings* identified in another 6 patients. SBFT was performed in 8 patients, with MD diagnosed in 5 patients and *significant other findings* in 1 patient. RBC scanning was performed in 4 patients, with no detection of MD or any *significant other findings*.

**Table 1:** Clinical characteristics of patients with adult bleeding Meckel’s diverticulum.

	Bleeding Meckel’s diverticulum (n = 35)
Demographics	
Age (years), mean ± S.D.	37.2 ± 15.7
< 40 years, n (%)	22 (62.9)
Male gender, n (%)	25 (71.4)
Clinical manifestation	
Overt small bowel bleeding, n (%)	27 (77.1)
Obscure small bowel bleeding, n (%)	8 (22.9)
Medical history	
Aspirin or NSAID use, n (%)	3 (8.6)
Comorbidity, n (%)	8* (17.1)
History of previous OGIB, n (%)	16 (45.7)
Laboratory results	
Hemoglobin (g/dL), mean ± S.D.	9.7±2.9
Hematocrit (%), mean ± S.D.	29.1±9.0
Platelet (1,000 cells/mm^3^), mean ± S.D.	219.9±868.5
PT (INR), mean ± S.D.	1.1±0.1
Surgery	
Laparoscopy *vs*. open laparotomy	
Laparoscopy, n (%)	13 (34.2)
Open laparotomy, n (%)	22 (65.8)
Type	
Small bowel resection and anastomosis, n (%)	28 (80.0)
Diverticulectomy, n (%)	7 (20.0)
Post-operative complications, n (%)	0 (0)
Pathology	
Ectopic tissue in diverticulum, n (%)	24 (68.6)
Gastric tissue, n (%)	22 (62.9)
Pancreatic tissue, n (%)	2 (5.7)
Prognosis	
Post-surgical symptom recurrence, n (%)	0 (0.0)

Abbreviation: NSAID, non-steroidal anti-inflammatory drugs; S.D., standard deviation.

*Hypertension (n = 2), type II diabetes (n = 2), schizophrenia (n = 1), chronic hepatitis B (n = 1), chronic hepatitis C (n = 1), previous chemotherapy due to diffuse large B cell lymphoma (n = 1)

### Diagnostic yields and accuracy of the different evaluation modalities for detecting adult bleeding MD

A priori, positive findings on Meckel’s scan were considered to be indicative of *certain* or *presumptive* MD. Therefore, both diagnostic yield and diagnostic accuracy for Meckel’s scan would be equal, with a calculated value of 21.4% (5.7%-51.2%). The diagnostic yield and accuracy of the other six modalities evaluated in our study are summarized in Table 2, with significance evaluated relative to the 21.4% values for Meckel’s scan. The following mean (95% CI) diagnostic accuracy was calculated for the six modalities: CE, 35.7% (14.0%-64.4%); BAE, 85.0% (61.1%-96.0%); mesenteric angiography, 0.0% (0.0%-80.2%); abdominal and pelvis CT, 31.8% (14.7%-54.9%); SBFT, 62.5% (25.9%-90.0%); and RBC scan, 0.0% (0.0%-60.4%). Therefore, BAE was the only diagnostic modality providing a significantly greater accuracy than Meckel’s scan (*P* = 0.001).

**Table 2:** The different modalities’ diagnostic accuracies and yields in the diagnosis of Meckel’s diverticulum in patients with obscure gastrointestinal bleeding.

	n	Certain or presumptive MD	Other significant findings*	Non-diagnostic	Diagnostic accuracy (95% CI)	*P*	Diagnostic yield (95% CI)	*p*
Meckel’s scan	14	3	0	11	21.4 (5.7–51.2)	referent	21.4 (5.7–51.2)	referent
Capsule endoscopy	14	5	6^¶^	3	35.7 (14.0–64.4)	0.675	78.6 (48.8–94.3)	0.008
Balloon-assisted enteroscopy	20	17	2^∫^	1	85.0 (61.1–96.0)	0.001	95.0 (73.1–99.7)	<0.001
Mesenteric angiography	2	0	1^§^	1	0.0 (0.0–80.2)	0.808	50.0 (2.7–97.3)	0.999
CT of the abdomen and pelvis	22	7	6^†^	9	31.8 (14.7–54.9)	0.766	59.1 (36.7–78.5)	0.061
Small bowel follow-through	8	5	1^‡^	2	62.5 (25.9–90.0)	0.142	75.0 (35.6–95.5)	0.045
RBC scan	4	0	0	4	0.0 (0.0–60.4)	0.801	0.0 (0.0–60.4)	0.801

Abbreviation: MD, Meckel’s diverticulum; Meckel’s scan, Technetium-99m pertechnetate scintigraphy; CT, computed tomography; RBC scan, Technetium-99m red blood cell scintigraphy.

* provided grounds to pursue further evaluation, but insufficient to diagnose to MD

^†^ileal wall thickening of unknown origin (n = 3), enteritis of unknown cause (n = 1), focal stricture (n = 1), obstruction (n = 1)

^‡^ulcers with pseudosacculation in the proximal ileum (n = 1)

^§^active bleeding from ileal branches of SMA (n = 1)

^¶^blood in lumen (n = 3), ulcerative lesions on the ileum (n = 2), Dieulafoy lesion (n = 1)

^∫^blood in lumen (n = 1), several ulcers in terminal ileum (n = 1)

The mean (95% CI) diagnostic yield for the respective evaluation modalities was as follows: CE, 50.0% (2.7%-97.3%); BAE, 95.0% (73.1%-99.7%); mesenteric angiography, 50.0% (2.7%-97.3%); abdominal and pelvis CT, 59.1% (36.7%-78.5%); SBFT, 75.0% (35.6%-95.5%); and RBC scan, 0.0% (0.0%-60.4%). Therefore, the diagnostic yield for CE, BAE, and SBFT was significantly higher than that for Meckel’s scan (*P* = 0.008, *P*<0.001, and *P* = 0.045, respectively).

### BAE in patients with bleeding MD

**Fig 1:**
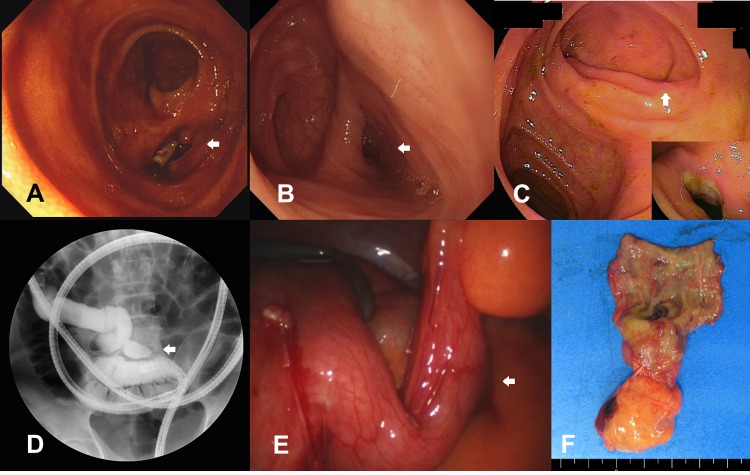
Meckel’s diverticulum (A-C) Balloon-assisted enteroscopy showed double-lumen signs and/or concurrent ulcerative lesions in the lumens (D) During balloon-assisted enteroscopy, about 4 cm sized luminal out-pouching diverticulum was identified in the distal ileum under fluoroscopy after radio-contrast dye injection, (E) During laparoscopy, about 4 cm sized diverticulum was identified, and (F) Surgical specimen of small bowel resection and anastomosis for Meckel’s diverticulum.

Of the 20 patients with bleeding MD who underwent BAE, double-balloon enteroscopy was performed in 16 patients and single-balloon enteroscopy in 4 patients. The procedures were performed using a retrograde approach in 15 patients, an anterograde approach in 1 patient, and using both retro- and anterograde approaches in 4 patients. The BAE successfully reached the MD in 17 of 20 cases. BAE failed in 3 cases due to the presence of strictures and massive bleeding. Of the 17 patients diagnosed with *certain* or *presumptive* MD on BAE, 15 MDs were localized <100 cm from the ICV, with another 2 localized at approximately 150 cm and 180 cm from the ICV. BAE revealed luminal diverticulae of approximately 2–4 cm in length, *with* or *without* ulceration or stricture, at the base of the small bowel (Fig 1). No BAE-related complications were identified.

### Clinical outcome of adult bleeding MD

Surgical follow-up from the evaluation was performed by laparoscopy in 13 patients and laparotomy in 22. Pathological analysis of the surgical MD specimens identified ectopic tissue in 24 patients (68.6%), gastric tissue in 22 patients, and pancreatic tissue in 2. No instances of postoperative complication or recurrence of symptoms were noted.

## Discussion

In clinical practice, MD is frequently suspected, often searched for, and seldom identified [13]. Among the 35 adults with bleeding MD evaluated at different centers, the diagnosis was missed in almost 30% of patients prior to surgical exploration. Accurate preoperative diagnosis of MD is important as it may lead to successful treatment of MD with minimally invasive surgery via laparoscopic procedures [14].

Meckel’s scan has traditionally been considered as the evaluative modality of choice for MD, based on its sensitivity of 85%, specificity of 95%, and overall accuracy of 90% in pediatric patients [3]. However, the diagnostic accuracy of Meckel’s scan has consistently been reported to be much lower for adults [1, 3, 4, 15]. In our study, we reported a diagnostic accuracy of Meckel’s scan for adults with bleeding MD of only 21%, indicative of the insufficient reliability of Meckel’s scan for the assessment of MD in adults. The false negative scans may be due to the rapid dilution of radioactive tracer secondary to rapid bleeding from the ectopic mucosa, impaired vascular supply, or insufficient gastric mucosa [13]. False negative scans may also result from hemorrhaging caused by the mechanical irritation of the bridge of tissue between the ileal lumen and the diverticulum [1].

In addition to Meckel’s scan, various radiological modalities have been used for the diagnosis of MD but such techniques are generally limited. As an example, MD is easily obscured by overlapping bowel loops in barium studies and by suboptimal small-bowel distention on CT [9, 16]. Mesenteric angiography and RBC scan can usually localize the locus of bleeding, but not necessarily identify its cause [9]. CE enables a visual examination of the entire small intestine; however, the lack of air insufflation and active manipulation, as well as active bleeding, can impair the detection of MD [17]. BAE resolves many of the limitations of these different techniques.

The mean insertion depth of retrograde BAE ranges between 100 and 150 cm, which theoretically, would be sufficient to identify the vast majority of MDs [6]. In our case series, the MD lesion was successfully reached using BAE in 17 of 20 cases (85.0%). Moreover, the diagnostic yield and accuracy of BAE were significantly higher than those for Meckle’s scan. Recently, He et al. reported a preoperative diagnostic yield of 86.5% for BAE carried out in 74 individuals with MD [18], a rate which is comparable with our results.

Small bowel bleeding should be considered in patients with gastrointestinal bleeding after performance of a normal upper and lower endoscopic examination. The etiology of small bowel bleeding is quite variable and CE should be considered as a first-line procedure for investigation. However, different evaluation modalities have been recommended depending on a patient’s status. For unstable patients with acute overt hemorrhaging, angiography should be performed on an emergent basis. In patients with occult hemorrhage or stable patients with active overt bleeding, CT enterography or angiography could be performed after CE to identify the source of the bleeding and to guide further management. In contrast to these procedures, BAE has not been routinely performed due to its invasiveness and cost, and limited expertise. However, it is important to consider that if a source of bleeding is identified in the small bowel and is associated with significant ongoing anemia and/or active bleeding during the diagnostic assessment phase, the patient should be managed with definite therapy, using endoscopy or surgery. If a source of bleeding is unidentified, then conservative management is typically recommended. Yet, the prognosis in these cases is generally poor, with repeated diagnostic investigations needed [19]. Therefore, when we consider that patients under the age of 40 years are more likely to have Meckel’s diverticulum, BAE should be considered.

Our study has several limitations which need to be acknowledged in the interpretation of results for practice. First, since the retrospective analysis of the patients only involved those with surgically confirmed MD, we could not include patients who had suspected MD but who actually had other conditions, which would provide a sample of false positive cases. This prevented the calculation of the sensitivity, specificity, and positive and negative predictive values of the diagnostic modalities being assessed. Furthermore, certain diagnostic modalities were performed on only a limited number of patients, which resulted in wide confidence intervals associated with the diagnostic yields. Second, despite this being a multicenter study, the sample size was small due to the rarity of bleeding MD in adults. Third, it is important to consider that the diagnostic yield and accuracy for CT and SBFT were relatively high in our study compared with those reported previously [9]. These higher than expected results for CT and SBFT likely reflect the stringent methods of our retrospective analysis protocol, with only confirmed cases of MD included. In addition, in our study, H2 receptor antagonist prior to Meckel’s scan was not injected, although the pharmacological intervention using H2 receptor antagonists has been reported to increase the sensitivity for the diagnosis of MD [20–22]. It is postulated that the release of pertechnetate from mucous cells and/or parietal cells into the diverticular lumen is delayed [23]. However, the exact mechanism that enhances visualization remains unclear and the usefulness of H2 receptor antagonist-enhanced 99m technetium pertechnetate imaging has not been evaluated in adult patients.

## Conclusions

Given the low diagnostic accuracy of Meckel’s scan within the adult population, BAE should be considered as a preferred preoperative diagnostic modality in adult patients who have small-bowel bleeding and a suspected diagnosis of MD. Therefore, BAE might be effective in avoiding repeated diagnostic assessments, extensive exploration, or intraoperative enteroscopy in adult patients with suspected MD.

## Abbreviations

MD: Meckel’s diverticulum
BAE: balloon-assisted enteroscopy
ICV: ileocecal valve
Meckel’s scan: Technetium-99m pertechnetate scintigraphy
CE: capsule endoscopy
BAE: balloon-assisted enteroscopy
CT: computed tomography
SBFT: small bowel follow-through
RBC scan: Technetium-99m red blood cell scintigraphy
95% CI: 95% confidence intervals
H2: histamine type-2
